# Fasting levels of growth hormone are associated with carotid intima media thickness but are not affected by fluvastatin treatment

**DOI:** 10.1186/s12872-017-0563-9

**Published:** 2017-05-16

**Authors:** Erik Hallengren, Peter Almgren, Maria Rosvall, Gerd Östling, Margaretha Persson, Andreas Bergmann, Joachim Struck, Gunnar Engström, Bo Hedblad, Olle Melander

**Affiliations:** 1 Department of Clinical Sciences, Lund University, Malmö, Sweden; 2 Department of Internal Medicine, Skåne University Hospital, Malmö, Sweden; 3 SphingoTec GmbH, Hohen Neuendorf, Germany; 4 Waltraut Bergmann Foundation, Hohen Neuendorf, Germany; 5 Department of Internal Medicine, SUS, Skåne University Hospital, Inga Marie Nilssons gata 36, SE 205 02 Malmö, Sweden

**Keywords:** Growth hormone, Statins, Imt, Lipids, Cardiovascular disease

## Abstract

**Background:**

Growth hormone (GH) has been linked to cardiovascular disease but the exact mechanism of this association is still unclear. We here test if the fasting levels of GH are cross-sectionally associated with carotid intima media thickness (IMT) and whether treatment with fluvastatin affects the fasting level of GH.

**Methods:**

We examined the association between GH and IMT in 4425 individuals (aged 46–68 years) included in the baseline examination (1991–1994) of the Malmö Diet and Cancer cardiovascular cohort (MDC-CC). From that cohort we then studied 472 individuals (aged 50-70 years) who also participated (1994–1999) in the β-Blocker Cholesterol-Lowering Asymptomatic Plaque Study (BCAPS), a randomized, double blind, placebo-controlled, single-center clinical trial. Using multivariate linear regression models we related the change in GH-levels at 12 months compared with baseline to treatment with 40 mg fluvastatin once daily.

**Results:**

In MDC-CC fasting values of GH exhibited a positive cross-sectional relation to the IMT at the carotid bulb independent of traditional cardiovascular risk factors (*p* = 0.002). In a gender-stratified analysis the correlation were significant for males (*p* = 0.005), but not for females (*p* = 0.09). Treatment with fluvastatin was associated with a minor reduction in the fasting levels of hs-GH in males (*p* = 0.05) and a minor rise in the same levels among females (*p* = 0.05).

**Conclusions:**

We here demonstrate that higher fasting levels of GH are associated with thicker IMT in the carotid bulb in males. Treatment with fluvastatin for 12 months only had a minor, and probably not clinically relevant, effect on the fasting levels of hs-GH.

**Electronic supplementary material:**

The online version of this article (doi:10.1186/s12872-017-0563-9) contains supplementary material, which is available to authorized users.

## Background

Recently we found that an increased fasting level of growth hormone (GH) is an independent predictor of cardiovascular morbidity and mortality [1]. This is somewhat surprising since GH in healthy adults is negatively associated with other predictors of cardiovascular disease (CVD) such as LDL-C, total cholesterol and triglycerides [1, 2]. GH is an anabolic stress hormone and a known regulator of lipid and glucose metabolism throughout the entire life [3]. One of the metabolic actions of GH is to increase the expression of hepatic LDL-receptors [4–6], which leads to reduced circulating levels of LDL-C. Concerning effects on glucose homeostasis, the actions of GH lead to insulin resistance and a deterioration of glucose tolerance [3, 7–9].

These two effects of GH, i.e. decreases LDL-C with negative effects on glucose homeostasis, is also seen with statins, which is one of the cornerstones in secondary and primary prevention of cardiovascular disease [10, 11]. Statins inhibit HMG-CoA-reductase, which leads to decreasing hepatic cholesterol concentration, up-regulation of LDL-receptors and eventually increased clearance of circulating low density lipoprotein cholesterol (LDL-C) [12, 13]. Similar to GH statins might have negative effects on glucose homeostasis and thus there is a small hazard of developing diabetes mellitus [14, 15]. In vitro studies also suggest that statins may lower GH gene expression [16].

Thus GH and statins share some effects on metabolism and we identified these similarities as an opportunity to further explore the previously discovered association between GH and CVD. We hypothesized that statins might affect the GH-concentration and measured the fasting levels of GH with a high-sensitivity assay (hs-GH) in a completed randomized controlled trial, originally designed to compare the effects of low-dose β-blockade and fluvastatin on the progression of carotid IMT during 36 months of treatment in subjects who had carotid plaque but no symptoms of carotid disease [17]. Since the relationship between fasting hs-GH and carotid IMT has not been previously described we also used a population based prospective cohort, the Malmö Diet and Cancer study cardiovascular cohort (MDC-CC), to study these variables. The objectives of our study were to investigate the relationship between fasting levels of GH and IMT and if treatment with fluvastatin affects the fasting level of hs-GH.

## Methods

### MDC-CC

The Malmö Diet and Cancer study – cardiovascular cohort (MDC-CC) is a prospective cohort examined 1991-96 with the aim to study the epidemiology of carotid artery disease. Further details about this study can be found in earlier publications [1, 18]. In brief participants underwent a physical examination and responded to a questionnaire about previous medical conditions, medications and life-style habits. Blood samples were drawn between 7.30 a.m. and 9.00 a.m. after an overnight fast and immediately stored at −80 °C. Measurement of hs-GH was made with a high-sensitivity chemiluminescence sandwich immunoassay (SphingoTec GmbH, Borgsdorf, Germany) previously described in detail [1]. The analytical assay sensitivity (mean relative light units of 20 determinations of GH free sample plus 2 S.D.; limit of detection, LOD) was 0.002 μg/L GH. The functional assay sensitivity (<20% inter assay CV; limit of quantification, LOQ) was 0.01 μg/L. GH concentration above the LOQ (0.01 μg/L) were measured with an interassay precision of typically below 10% CV. The assay was calibrated using dilutions of GH (NIBSC code 98/574, National Institute for Biological Standards and Control, Herfordshire, UK).

### Carotid ultrasound

Carotid B-mode ultrasound was performed by the same 2 trained and certified sonographers at the baseline examination in the MDC-CC and in the BCAPS at baseline, 18 and 36 months as previously described [17, 19]. The same methodology was used in both studies. In brief the mean wall thickness in the right common carotid artery 1 cm proximal to the bifurcation was measured according to the leading-edge principle (IMT_mean_CCA). The bifurcation area of the right common carotid artery was further scanned within a predefined window comprising 3 cm of the distal common carotid artery, the bulb, and 1 cm of the internal and external carotid artery, respectively, for the occurrence of plaques and measured as the maximum IMT at the bifurcation (IMT_max_Bulb). At regular intervals (<3 weeks) during the ultrasound investigation procedure, intra- and interobserver variation analyses were performed. The mean absolute difference between two measurements in percentage with one observer measuring carotid IMT was 9.0 (standard deviation, 7.2) percent (*r* = 0.77) and, when using two observers, was 8.7 (standard deviation, 6.2) percent (*r* = 0.85). Corresponding values for the measurements of carotid stenosis assessed by Kendall’s tau were as follows: τ = 0.65 and τ = 0.72, respectively [19].

### Cross-sectional analysis of IMT and hs-GH in MDC-CC

We performed a cross-sectional analysis of the relationship between hs-GH and common carotid artery (CCA) IMT_mean_ and bifurcation (Bulb) IMT_max_, in the MDC-CC. In multivariate regression models with IMT_mean_CCA as the dependent variable and the standardized value of the natural logarithm of hs-GH as independent we analyzed 4425 subjects (aged 46–68 years; 58% women) with values on both hs-GH and IMT_mean_CCA. One model was adjusted for age and sex, and a second model was in addition adjusted for a set of traditional cardiovascular risk factors: smoking, systolic blood pressure, anti-hypertensive medication, body mass index (BMI), LDL-C, high density lipoprotein cholesterol (HDL-C) and diabetes mellitus. In females an additional analysis was performed adjusted for menopausal status (pre, peri, post) and use of hormone replacement therapy.

The same approach was used in the analysis of hs-GH and IMT_max_Bulb, where 3397 subjects (aged 46–68 years, 58% women) had complete data.

### BCAPS

The β-Blocker Cholesterol-Lowering Asymptomatic Plaque Study (BCAPS) was a randomized, double blind, placebo-controlled, single-center clinical trial that took place between 1994 and 1999. A detailed description of BCAPS can be found in the original paper [17], following here is a brief summary.

**Fig. 1:**
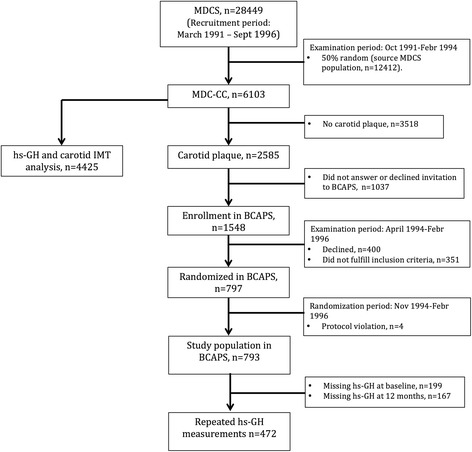
Flow chart of the recruitment to MDC-CC and BCAPS. Overview of the recruitment to the study populations from the Malmö Diet and Cancer study. Figure shows the relation between the studies and the times and reasons for exclusion. Abbreviations: MDCS – Malmö Diet and Cancer study

The original study population consisted of 361 men and 432 women (total *n* = 793) 49 to 70 years of age recruited from the MDC-CC (Fig. 1). All participants included in BCAPS had carotid plaque but no symptoms of carotid artery disease. Exclusion criteria were: myocardial infarction, angina pectoris or stroke within the previous 3 months; previous surgery in the right carotid artery; regular use of β-blockers or statins; blood pressure > 160 (systolic) or >95 (diastolic) mm Hg; total cholesterol >8.0 mmol/l; hyperglycemia suspected to require insulin treatment; and conditions that in the view of the investigator made the participant unsuitable for the trial.

### Randomization

**Fig. 2:**
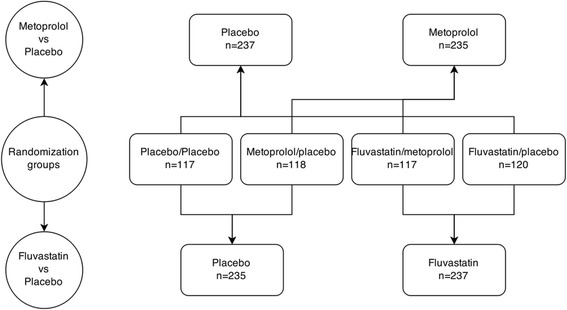
Randomization groups in BCAPS. Schematic drawing of the randomization groups in BCAPS

The participants were randomly assigned to 1 of 4 drug combination groups according to a factorial design (Fig. 2): placebo/placebo, metoprolol CR/XL (25 mg once daily)/placebo, fluvastatin (40 mg once daily)/placebo or metoprolol CR/XL (25 mg once daily)/fluvastatin (40 mg once daily).

### Study length and blood sampling in BCAPS

The treatment period was 36 months. Blood samples were drawn after an overnight fast between 7.30 a.m. and 10.00 a.m. at baseline and at 12, 24 and 36 months. Levels of hs-GH were measured in stored fasting plasma samples, which were frozen immediately to −20 °C at the different examinations. The same high-sensitivity immunoassay used for measuring hs-GH in MDC-CC was used to measure hs-GH in BCAPS. A number of the samples stored in the freezer were missing or had insufficient plasma for the analysis of hs-GH. Subjects in which either the baseline-value of hs-GH (*n* = 199) or the 12-month value of hs-GH (*n* = 167) was missing were excluded from further analysis in BCAPS. After this adjustment the study consisted of 472 individuals (aged 50-70 years; 63% women).

### Statistical analyses in BCAPS

The four original randomization groups are in some analyses pooled into fluvastatin vs non-fluvastatin (i.e placebo) or metoprolol vs non-metoprolol (Fig. 2). Since fasting hs-GH is known to differ between men and women all models were performed separate for men and women as well as combined.

To investigate if fasting hs-GH was affected by treatment with fluvastatin or metoprolol we performed multivariate linear regression analysis with the standardized value of the natural logarithm of ΔGH (baseline value subtracted from 12 month value) as the dependent variable and the treatment group as an independent variable and adjusted for sex, age, and the natural logarithm of the fasting level of hs-GH at baseline. Separate models were performed with each of the treatment groups vs the original or pooled placebo groups depending on model. As a sensitivity analysis another model was in addition adjusted for LDL-C at baseline and at 12 months.

We then analyzed if the baseline value of, or 12-month change in, hs-GH affects the treatment effect of fluvastatin or metoprolol on the IMT at 36 months in a series of multivariate regression models. A small number of individuals did not complete the ultrasound examination at 36 months and in these individuals the latest IMT-values (i.e. at 18 months) are used. The analyses were adjusted for time between IMT-measurements, IMT at baseline, age, sex (if not already gender separated) and the natural logarithm of the fasting level of hs-GH at baseline.

The initial model analyzed IMT at 36 months as the dependent variable and hs-GH at baseline and treatment with fluvastatin or metoprolol as independent variables. If the main effects (GH and medication) were significant we would then perform an interaction analysis.

The second model analyzed IMT at 36 months as outcome, but with ΔGH (0–12 months) as the independent variable together with treatment with fluvastatin or metoprolol.

All analyses were performed in SPSS (version 22.0.0, SPSS Inc., Chicago, Ill). A 2-sided *P*-value of less than 0.05 was considered statistically significant.

## Results

### Association between carotid IMT and hs-GH in MDC-CC

Baseline characteristics of the MDC-CC have been published previously [1]. In the cross-sectional linear regression model of 3397 (1957 females, 58%) individuals, the fasting levels of hs-GH exhibited a significant positive correlation with IMT_max_Bulb (*P* = 0.003) (Table 1). In a sex-stratified analysis this positive association was significant in males (*P* = 0.003) but not among females (*P* = 0.18). The associations among males and the total cohort remained strong when adjusting for traditional cardiovascular risk factors (*P* = 0.002 and *P* = 0.005 respectively).

**Table 1:** Cross-sectional linear regression in the MDC-CC with IMT at baseline as dependent variable and hs-GH as independent. One crude model adjusted for sex and age and one model in addition adjusted for traditional cardiovascular risk factors^a^

Dependent	Gender	Model^a^	Beta^b^	95%CI	*P*
IMT_max_ Bulb	ALL	Crude	0.060	0.020 to 0.099	0.003
	(*n* = 3397)	Adjusted	0.066	0.025 to 0.106	0.002
	MALE	Crude	0.075	0.025 to 0.125	0.003
	(*n* = 1440)	Adjusted	0.074	0.022 to 0.125	0.005
	FEMALE	Crude	0.029	−0.014 to 0.072	0.18
	(*n* = 1957)	Adjusted	0.038	−0.006 to 0.083	0.09
IMT_mean_CCA	ALL	Crude	−0.012	−0.046 to 0.021	0.48
	(*n* = 4425)	Adjusted	0.017	−0.017 to 0.052	0.32
	MALE	Crude	0.011	−0.033 to 0.054	0.63
	(*n* = 1767)	Adjusted	0.024	−0.020 to 0.068	0.28
	FEMALE	Crude	−0.027	−0.063 to 0.009	0.15
	(*n* = 2658)	Adjusted	0.001	−0.036 to 0.038	0.97

^a^Crude models adjusted for sex and age. Adjusted models adjusted for: sex, age, systolic blood pressure, antihypertensive medication, diabetes mellitus, current smoking, BMI, LDL-C and HDL-C

^b^The β coefficients are expressed as the increment of standardized values of the natural logarithm of IMT per 1 increment of standardized values of the natural logarithm of hs-GH

No significant associations were found between the fasting levels of hs-GH and IMT_mean_CCA neither crude nor adjusted in the 4425 individuals (2658 females, 60%) that were available in this analysis.

To rule out that the difference in the results with the association with hs-GH between the two IMT measurements were not due to a selection bias, we also analyzed IMT_mean_CCA in the 3397 individuals mentioned previously. IMT_mean_CCA was available in 3390 of these and did not show any significant correlation with hs-GH in crude and adjusted models (data not shown).

As sensitivity analyses the adjusted regression models were additionally adjusted for variables that could affect GH. The relation between hs-GH and IMT in women remained non-significant after additional adjustment for menopausal status and hormone replacement therapy. Additional adjustment for waist hip ratio (WHR) marginally improved all models. The results in IMT_mean_CCA were however still non-significant, but adjustment for WHR in IMT_max_Bulb made fasting hs-GH significantly associated with IMT_max_Bulb in females (Beta, 0.044; 95%CI, 0.000-0.089; *P* = 0.05) and the association remained strong among all (Beta, 0.068; 95%CI, 0.027–0.108; *P* = 0.001) and males (Beta, 0.074; 95%CI, 0.022–0.125; *P* = 0.005).

### BCAPS

Baseline characteristics of the 472 individuals in the BCAPS study population were similar in the different treatment groups and are shown in Table 2. The females in all groups had higher values of hs-GH at baseline compared with males. In the group of subjects in which hs-GH could not be measured due to missing samples at either baseline or 12 months there were more males than females (Additional file 1: Table S1). The median values for IMT_mean_CCA and IMT_max_Bulb were higher in the males excluded from the study, while the anthropometrics were similar to the study population in both genders. The missing samples were evenly distributed over the different treatment groups but the male/female ratio was marginally different in two of the four treatment groups (Table 2).

**Table 2:** Baseline clinical characteristics of the study population in BCAPS

Variable	Placebo/Placebo	Metoprolol/Placebo	Fluvastatin/Metoprolol	Fluvastatin/Placebo
Number of participants	117	118	117	120
Female (%)	78 (66.7)	78 (66.1)	70 (59.8)	72 (60.0)
Age, mean (SD), years	61.5 (5.7)	60.3 (5.6)	62.3 (5.0)	61.8 (5.4)
Height, mean (SD), cm	168 (10)	167 (8)	169 (8)	169 (9)
Body Mass Index, Mean (SD), kg/m2	25.5 (3.6)	25.5 (3.6)	25.1 (2.7)	25.7 (3.7)
LDL-C, mean (SD), mmol/L	4.07 (0.86)	4.18 (0.92)	4.15 (0.88)	4.16 (0.82)
HDL-C, mean (SD), mmol/L	1.48 (0.40)	1.39 (0.37)	1.41 (0.35)	1.37 (0.35)
IMT_mean_ CCA, median (IQR)	0.88 (0.78-0.96)	0.88 (0.77-0.99)	0.86 (0.79-0.98)	0.86 (0.78-0.97)
IMT_max_ Bulb median (IQR)	1.74 (1.46-2.09)	1.77 (1.49-2.24)	1.84 (1.52-2.29)	1.81 (1.55-2.17)
Growth Hormone - males, median (IQR), μg/L	0.28 (0.06-0.84)	0.15 (0.06-0.35)	0.15 (0.06-0.97)	0.18 (0.08-0.57)
Growth Hormone - females, median (IQR), μg/L	1.49 (0.58-3.14)	1.52 (0.49-2.64)	1.16 (0.53-2.32)	1.53 (0.68-2.74)

Missing values in IMT_max_bulb: placebo/placebo, *n* = 3; metoprolol/placebo *n* = 3; Fluvastatin/Metoprolol, *n* = 5; fluvastatin/placebo, *n* = 8

Missing values in HDL-C and LDL-C: placebo/placebo, *n* = 1; metoprolol/fluvastatin *n* = 1

### hs-GH and treatment with fluvastatin

**Fig. 3:**
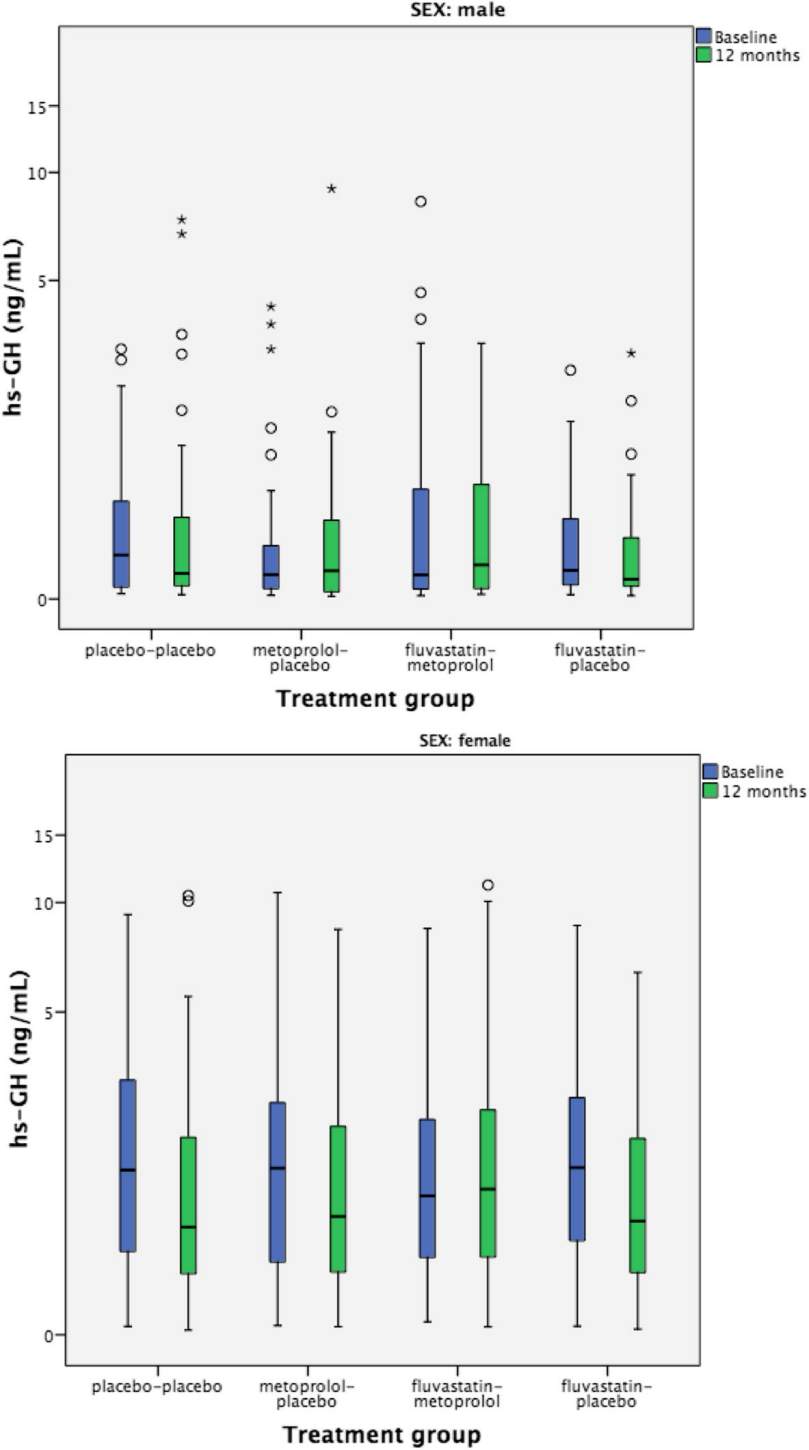
Boxplots of fasting levels of hs-GH in the different randomization groups at baseline and after 1 year of treatment

Unadjusted values in the treatment groups on hs-GH and IMT at follow-up can be found in Table 3 and Fig. 3. In linear regression models the change in levels of hs-GH at 12 months compared with baseline (ΔGH) were related against the different treatment groups (Table 4). In males treatment with fluvastatin/metoprolol was associated with a reduction of hs-GH over 12 months compared with placebo (*P* = 0.03). The levels of hs-GH were also reduced over 12 months with fluvastatin treatment compared with placebo in males (*P* = 0.05). On the contrary, among females both treatment with fluvastatin/metoprolol and fluvastatin was associated with an increase in the level of hs-GH over 12 months (*P* = 0.02 and *P* = 0.05 respectively) when compared with placebo.

**Table 3:** Characteristics of the BCAPS population after medical treatment

Variable	Placebo/Placebo	Metoprolol/Placebo	Fluvastatin/Metoprolol	Fluvastatin/Placebo
12 months				
LDL-C, mean (SD), mmol/L	4.01 (0.82)	4.17 (0.87)	3.21 (0.85)	3.27 (0.74)
Growth Hormone - males, median (IQR), μg/L	0.16 (0.08-0.59)	0.17 (0.04-0.57)	0.21 (0.06-0.95)	0.12 (0.07-0.44)
Growth Hormone - females, median (IQR), μg/L	0.82 (0.40-1.99)	0.93 (0.41-2.20)	1.25 (0.53-2.50)	0.88 (0.41-2.01)
36 months				
IMT_mean_ CCA, median (IQR)	0.91 (0.82-1.04)	0.90 (0.83-1.02)	0.87 (0.78-0.98)	0.87 (0.77-0.99)
IMT_max_ Bulb, median (IQR)	1.98 (1.71-2.35)	1.89 (1.60-2.43)	1.99 (1.60-2.44)	2.02 (1.68-2.30)

Missing values in IMT_mean_ CCA: placebo/placebo, *n* = 4; metoprolol/placebo *n* = 1; fluvastatin/placebo, *n* = 1

Missing values in IMT_max_ Bulb: placebo/placebo, *n* = 6; metoprolol/placebo *n* = 4; Fluvastatin/Metoprolol, *n* = 6; fluvastatin/placebo, *n* = 8

Missing values in LDL-C: placebo/placebo, *n* = 2; metoprolol/placebo *n* = 6; Fluvastatin/Metoprolol, *n* = 1; fluvastatin/placebo, *n* = 3

**Table 4:** Multivariate linear regression models of the effect of different treatment regimes on the change in fasting levels of hs-GH over 12 months in BCAPS

Sex	Model^a^	Treatment group	N (med/placebo)	B^b^	95%CI	*P*
All	Original	Metoprolol-Placebo	118/117	−0.09	−0.35 to 0.17	0.48
		Fluvastatin-Metoprolol	117/117	0.10	−0.13 to 0.33	0.38
		Fluvastatin-Placebo	120/117	−0.04	−0.24 to 0.17	0.72
	Pooled	Fluvastatin	237/235	0.08	−0.09 to 0.24	0.24
	Pooled	Metoprolol	235/237	0.02	−0.14 to 0.19	0.80
Male	Original	Metoprolol-Placebo	40/39	−0.17	−0.55 to 0.21	0.38
		Fluvastatin-Metoprolol	47/39	−0.53	−1.02 to −0.04	0.03
		Fluvastatin-Placebo	48/39	−0.15	−0.42 to 0.11	0.26
	Pooled	Fluvastatin	95/79	−0.27	−0.54 to 0.00	0.05
	Pooled	Metoprolol	87/87	−0.25	−0.52 to 0.02	0.07
Female	Original	Metoprolol-Placebo	78/78	−0.07	−0.38 to 0.25	0.68
		Fluvastatin-Metoprolol	70/78	0.32	0.06 to 0.58	0.02
		Fluvastatin-Placebo	72/78	0.03	−0.23 to 0.28	0.83
	Pooled	Fluvastatin	142/156	0.20	0.00 to 0.41	0.05
	Pooled	Metoprolol	148/150	0.10	−0.10 to 0.31	0.33

Models adjusted for: age and standardized values of natural logarithm of GH at baseline. In addition adjusted for sex in the analysis for all

^a^Three different models are performed: in “original” the different treatment groups are each one compared with placebo. In “pooled” the individuals receiving fluvastatin are pooled and compared with individuals not receiving fluvastatin and vice versa with metoprolol.

^b^B coefficients are expressed as the SD increment of the natural logarithm of ΔGH (12 months – baseline) with treatment of the medicine in question as compared with placebo.

When analyses were in addition adjusted for levels of LDL-C at baseline and at 12 months, the results were attenuated so that none of the treatments were associated with the fasting levels of hs-GH (Additional file 1: Table S2).

### hs-GH and effect of medical therapy on the IMT outcome in BCAPS

Multivariate regression models including hs-GH at baseline and the pooled randomization groups with IMT outcome at 36 months as dependent variable were performed to evaluate if hs-GH at baseline affected the outcome of medical therapy on the IMT. Adjusted for treatment with fluvastatin, the baseline fasting value of hs-GH did not show any associations with neither IMT_mean_CCA nor IMT_max_Bulb outcome in the whole cohort (*P* = 0.75 and *P* = 0.54 respectively), in males (*P* = 0.95 and *P* = 0.76) and in females (*P* = 0.54 and *P* = 0.36). When instead adjusting for treatment with metoprolol the correlations between the fasting value of hs-GH and IMT_mean_CCA or IMT_max_Bulb outcome were still non-significant in the whole cohort (*P* = 0.74 and *P* = 0.48 respectively), in males (*P* = 0.96 and *P* = 0.79) and in females (*P* = 0.59 and *P* = 0.28).

In similar multivariate regression models as in the previous paragraph we investigated if ΔGH (12-0 months) was associated with IMT outcome at 36 months, to evaluate if change in hs-GH affected the outcome of medical therapy on the IMT. ΔGH was not associated with neither IMT_mean_CCA nor IMT_max_Bulb outcome when adjusting for treatment with fluvastatin in the whole cohort (*P* = 0.74 and *P* = 0.18 respectively), in males (*P* = 0.98 and *P* = 0.39) and in females (*P* = 0.67 and *P* = 0.48). When instead adjusting for treatment with metoprolol the correlations between ΔGH and IMT_mean_CCA or IMT_max_Bulb outcome were still non-significant in the whole cohort (*P* = 0.64 and *P* = 0.16 respectively), in males (*P* = 0.60 and *P* = 0.33) and in females (*P* = 0.51 and *P* = 0.34).

Since the correlations between GH or change in GH and IMT outcome at 36 months in BCAPS were non-significant, no further interaction analyses between hs-GH and the different medical treatments were performed.

## Discussion

This is the first study examining whether the fasting levels of hs-GH are associated with carotid IMT in the general population and if treatment with fluvastatin affects the fasting values of hs-GH. Higher fasting levels of hs-GH were associated with an increased IMT in the carotid bulb in males independently of traditional cardiovascular risk factors. The fasting levels of hs-GH were only to a minor, and probably clinically non-relevant, degree affected by treatment with fluvastatin.

### Fasting hs-GH and IMT

Previous results have shown that higher fasting values of hs-GH are associated with increased cardiovascular morbidity and mortality, especially in males [1, 20]. The positive association between hs-GH and the IMT at the carotid bulb in the current study is in harmony with these former results and the relation is once again stronger in the male part of the population. As atherosclerosis primarily presents in the bulb region, the IMT in the carotid bulb is a good measure of atherosclerosis and has been shown to be useful in risk prediction [21]. The IMT in the common carotid artery is more associated with hypertension and stroke [21], i.e. vascular aging and did not show any association with fasting hs-GH in our study. This suggests that the previously described link between fasting levels of hs-GH and cardiovascular disease could be mediated by atherosclerosis, at least in males.

Several studies have been made to elucidate the connection between IMT and GH in the more extreme forms of GH deficiency or GH excess (acromegaly). Results are often conflicting and comparisons between the studies are difficult since different measurements of IMT often are used. In line with our results, a study on acromegalic patients showed increased IMT (composite variable of bulb + CCA) in patients when compared with controls [22]. At the other end, GH-deficient patients have been shown to have an elevated mean bulb IMT when compared with controls, but not when compared with BMI-matched controls [23, 24]. Bulow et. al investigated the bulb IMT in female GH-deficient patients and did not find any significant difference when compared with healthy controls [25]. GH replacement therapy has in some studies shown beneficial effects on the bulb IMT in individuals with adult-onset GHD [23, 26, 27], but negative effects in congenital GHD [28]. Thus, data from GHD-patients are conflicting and if anything indicates a negative association between GH and bulb IMT, but data from acromegaly patients support our finding with the association being positive. It is however uncertain to what extent data from these previous studies can be extrapolated to a general healthy population, such as ours.

In our previous study, where we found that the fasting levels of hs-GH independently predicted cardiovascular disease and death, we observed that this association was to a larger extent driven by the male part of the population [1]. In the current study we observed similar gender differences, hs-GH was associated with thickness of the carotid bulb only in men, which is not surprising given our previous results. One explanation of this difference might be that women have higher basal levels and a more irregular secretion of GH than men [1, 29, 30]. This probably makes a single hs-GH value a more uncertain measure in women, even if drawn fasting in the morning.

### Effect of fluvastatin treatment on fasting hs-GH

In males, treatment with metoprolol/fluvastatin compared with placebo/placebo was associated with a reduction of hs-GH over 12 months of treatment independently of baseline level of hs-GH. The effect was however quite small and the change in absolute values were minor. It should also be noted that the change of hs-GH over 12 months in the fluvastatin/placebo-group was not significantly different when compared with the placebo/placebo group. The same applies to the female part of the cohort where treatment with metoprolol/fluvastatin and fluvastatin (pooled) was associated with elevation of hs-GH over 12 months. The elevation of hs-GH among women was not absolute, but relative as the placebo/placebo group had the greatest reduction in levels of fasting hs-GH (Fig. 3).

The pharmacokinetics of fluvastatin in men and women are not known to differ substantially [31]. Two recent studies investigating gender differences on statin treatment, with regards to its cholesterol lowering and cardio-protective effects, did not find any large differences that could explain our results [32, 33]. Possibly the irregular secretion of hs-GH in females (see previous section) could have influenced the data.

When adjusting for LDL-C at baseline and at 12 months the associations between treatment and fasting hs-GH were no longer significant in any gender. Taken together, our data indicate that any effects of fluvastatin or metoprolol on the fasting levels of hs-GH are small and probably not clinically relevant.

### Strengths and limitations

There are some limitations to our study. First, the original randomized clinical trial was neither designed nor purposed to analyze the effect on the fasting level of hs-GH. About 40% of the participants had insufficient amount of plasma stored in the freezer from either the examination at baseline or at 12 months and we were forced to exclude them to keep an intact study population. This probably makes the fluvastatin-part of the study a bit underpowered. A larger part of the missing samples were drawn from male participants and both measures of the IMT in these males were higher than the IMT of the males in the study. A selection bias cannot be ruled out, but we do not have a plausible theory of the mechanism of this selection. The missing samples made the male/female ratio different in the treatment groups, but as all analyses were performed separate for men and women, this should not be an issue.

We chose to focus our analysis on the change in hs-GH after 12 months of treatment. The reasons for this are many. First, we hypothesized that any change in the levels of hs-GH would be visible at this time and that any potential effect of the medicine on hs-GH would not increase with a longer treatment period. Second, the data at 12 months is probably the most “clean” data, compliance tend to be highest in the beginning of a study. Third, some subjects were during follow-up prescribed open-label lipid lowering therapy, antihypertensive medication etc. [17], which also might affect the data more at later time points. Finally, there were missing plasma samples at all time points and if we would extend our analysis to them we would have been forced to exclude additional individuals and thus further diminishing our study population.

The pulsatile mode of secretion of hs-GH is as previously discussed [1] a limitation since the blood samples might be taken during a peak in a few individuals. The samples were drawn in the morning when peaks are scarce [34–36] and this potential irregularity in the measurements would, if anything, dilute an association. A single fasting value of hs-GH is not a standard clinical test, the correlation between fasting GH and 24-h GH secretion is strong, but with large variability [37]. However as we have previously shown on a population basis [1], a single fasting value of hs-GH strongly and independently predicts cardiovascular disease and death, which calls for further research on the subject. Apart from the pulsatile GH release, it should be emphasized that there are many potential external influences on fasting GH such as emotional or physical stress and that this may dilute any true relationship between hs-GH and carotid IMT.

## Conclusions

In conclusion we here demonstrate that the fasting levels of hs-GH are associated with the IMT at the carotid bulb in males and suggest atherosclerosis as the likely cause of the previously described association between hs-GH and hard cardiovascular endpoints. Treatment with fluvastatin was associated with only small changes, which differed between the genders, in the fasting levels of hs-GH. This indicates that any effect of fluvastatin on fasting hs-GH is small and probably not clinically relevant.

## Additional file

Additional file 1: Table S1. Clinical characteristics of participants not included in the analysis due to missing blood samples. **Table S2**. Multivariate linear regression models of the effect of different treatment regimes on the change in fasting levels of hs-GH over 12 months in BCAPS. Adjusted for LDL-C at baseline and at 12 months. (DOCX 16 kb)

## Abbreviations

BCAPS: The β-blocker cholesterol-lowering asymptomatic plaque study
GH: Growth hormone
hs-GH: High sensitivity growth hormone
IMT: Intima media thickness
IMT_max_Bulb: Maximum Intima Media Thickness in the carotid bulb
IMT_mean_CCA: Mean Intima Media Thickness in the common carotid artery
MDC-CC: Malmö diet and cancer study – cardiovascular cohort
