# Supplementary material for: Fasting levels of growth hormone are associated with carotid intima media thickness but are not affected by fluvastatin treatment
Source: BMC Cardiovasc Disord. 2017 May 16;17:125. doi: 10.1186/s12872-017-0563-9 (PMC5434616; doi:10.1186/s12872-017-0563-9)
Supplement: Additional file 1: Table S1. — Clinical characteristics of participants not included in the analysis due to missing blood samples. Table S2. Multivariate linear regression models of the effect of different treatment regimes on the change in fasting levels of hs-GH over 12 months in BCAPS. Adjusted for LDL-C at baseline and at 12 months. (DOCX 16 kb) [file 12872_2017_563_MOESM1_ESM.docx]

| **Table S1**: Clinical characteristics of participants not included in the analysis due to missing blood samples. | | | | |
| --- | --- | --- | --- | --- |
| **Variable** | **Included (n=472)** |  | **Missing (n=320)** |  |
| **Sex** | **Male** | **Female** | **Male** | **Female** |
| Number (% of group) | 174 (37) | 298 (63) | 187 (58) | 133(42) |
| Age, mean (SD), years | 61.8 (5.5) | 61.3 (5) | 62.2 (5.1) | 62.1 (5.3) |
| Body Mass Index, Mean (SD), kg/m2 | 25.6 (3.3) | 25.3 (3.5) | 25.8 (3.3) | 25.5 (4.4) |
| LDL-C, mean (SD), mmol/L | 4.13 (0.86) | 4.15 (0.87) | 4.11 (0.89) | 4.18 (0.93) |
| HDL-C, mean (SD), mmol/L | 1.25 (0.28) | 1.51 (0.38) | 1.22 (0.31) | 1.47 (0.38) |
| IMTmean CCA: at baseline, median (IQR) | 0.88 (0.79-1.00) | 0.86 (0.77-0.95) | 0.91 (0.79-1.02) | 0.86 (0.78-0.95) |
| IMTmax Bulb at baseline, median (IQR) | 1.82 (1.55-2.27) | 1.78 (1.48-2.18) | 1.94 (1.55-2.37) | 1.75 (1.45-2.08) |
| Height, mean (SD), cm | 177 (6) | 164 (6) | 176 (7) | 164 (6) |
|  |  |  |  |  |
| Abbreviations: LDL-C, Low-density lipoprotein cholesterol; HDL-C, High-density lipoprotein cholesterol | | | | |
| Individuals available for analysis: LDL-C: Included males n= 173, included females n=297, excluded males n=181, excluded females n=130. | | | | |
| Individuals available for analysis: HDL-C: Included males n= 173, included females n=297, excluded males n=184, excluded females n=131. | | | | |
| Individuals available for analysis: IMTmax Bulb: Included males n= 167, included females n=286, excluded males n=178, excluded females n=126. | | | | |

**Table S2**: Multivariate linear regression models of the effect of different treatment regimes on the change in fasting levels of hs-GH over 12 months in BCAPS. Adjusted for LDL-C at baseline and at 12 months.

| **Sex** | **Model*** | **N(med/pla)** | **Treatment group** | **B**† | **95%CI** | **P** |
| --- | --- | --- | --- | --- | --- | --- |
| All | Original | 112/114 | Metoprolol-Placebo | -0.10 | -0.38 to 0.17 | 0.45 |
| (n=459) |  | 116/114 | Fluvastatin-Metoprolol | 0.10 | -0.19 to 0.38 | 0.52 |
|  |  | 117/114 | Fluvastatin-Placebo | -0.04 | -0.30 to 0.22 | 0.77 |
|  | Pooled | 233/226 | Fluvastatin | 0.06 | -0.15 to 0.27 | 0.59 |
|  | Pooled | 228/231 | Metoprolol | 0.02 | -0.15 to 0.19 | 0.83 |
| Male | Original | 40/38 | Metoprolol-Placebo | -0.16 | -0.56 to 0.24 | 0.43 |
| (n=172) |  | 46/38 | Fluvastatin-Metoprolol | -0.47 | -1.10 to 0.17 | 0.15 |
|  |  | 48/38 | Fluvastatin-Placebo | -0.15 | -0.50 to 0.19 | 0.38 |
|  | Pooled | 94/78 | Fluvastatin | -0.22 | -0.57 to 0.13 | 0.21 |
|  | Pooled | 86/86 | Metoprolol | -0.26 | -0.53 to 0.02 | 0.070 |
| Female | Original | 72/76 | Metoprolol-Placebo | -0.07 | -0.41 to 0.26 | 0.67 |
| (n=287) |  | 70/76 | Fluvastatin-Metoprolol | 0.27 | -0.06 to 0.60 | 0.11 |
|  |  | 69/76 | Fluvastatin-Placebo | 0.01 | -0.31 to 0.33 | 0.95 |
|  | Pooled | 139/148 | Fluvastatin | 0.15 | -0.11 to 0.41 | 0.26 |
|  | Pooled | 142/145 | Metoprolol | 0.10 | -0.11 to 0.31 | 0.35 |

*Three different models are performed: in "original" the different treatment groups are each one compared with placebo. In "pooled" the individuals receiving fluvastatin are pooled and compared with individuals not receiving fluvastatin and vice versa with metoprolol.

†B coefficients are expressed as the SD increment of the natural logarithm of ΔGH (12 months – baseline) with treatment of the medicine in question as compared with placebo.

Models adjusted for: age, LDL-C at baseline, LDL-C at 12 months, and standardized values of natural logarithm of GH at baseline. In addition adjusted for sex in the analysis for all.
